# Uncovering Overlapping Gene Networks and Potential Therapeutic Targets in Osteoporosis and COVID-19 Through Bioinformatics Analysis

**DOI:** 10.1155/ije/8816596

**Published:** 2025-08-30

**Authors:** Yuwen Luo, Shizhen Liu, Xianyin Liu, Shu Zhong, Ye Wang, Zheng Wan

**Affiliations:** ^1^Department of Orthopaedics, Dongguan Hospital Affiliated to Southern Medical University (Dongguan People's Hospital), Dongguan 523059, Guangdong, China; ^2^Department of Geriatrics, Zhongshan Hospital Xiamen University, School of Medicine, Xiamen University, Xiamen 361000, Fujian, China

**Keywords:** bioinformatics, biomarkers, COVID-19, osteoporosis, therapeutic targets

## Abstract

**Background:** Osteoporosis is a progressive bone disease characterized by reduced bone density and deterioration of bone microarchitecture, predominantly affecting the elderly population. The ongoing COVID-19 pandemic has introduced additional challenges in osteoporosis management, potentially due to systemic inflammation and direct viral impacts on bone metabolism. This study aims to identify common differentially expressed genes (DEGs) and key molecular pathways shared between osteoporosis and COVID-19, with the goal of uncovering potential therapeutic targets through bioinformatics analysis.

**Methods:** Publicly available gene expression datasets GSE164805 (osteoporosis) and GSE230665 (COVID-19) were analyzed to identify overlapping DEGs. Functional enrichment analysis using Gene Ontology (GO), pathway analysis, protein–protein interaction (PPI) network construction, and transcription factor (TF)–hub gene regulatory network analysis were performed to explore the biological significance and regulatory mechanisms of these DEGs.

**Results:** A total of 325 common DEGs were identified between osteoporosis and COVID-19. GO enrichment analysis revealed significant involvement in signal transduction and plasma membrane components. Pathway analysis highlighted the “cytokine–cytokine receptor interaction” pathway as a central player. PPI network analysis identified a module of 193 genes with 397 interactions, from which 10 key hub genes were prioritized: ACTB, CDH1, RPS8, IFNG, RPL17, UBC, RPL36, RPS4Y1, GSK3B, and FGF13. Furthermore, 76 TFs were found to regulate these hub genes, and 15 existing drugs targeting four of these hub genes were identified.

**Conclusion:** This integrative bioinformatics study reveals 15 candidate therapeutic agents that target key regulatory genes shared between osteoporosis and COVID-19, offering promising treatment strategies for osteoporotic patients, especially those impacted by or at risk of SARS-CoV-2 infection.

## 1. Introduction

Osteoporosis is a progressive metabolic bone disease characterized by reduced bone mineral density (BMD) and deterioration of bone microarchitecture, resulting in increased bone fragility and a higher risk of fractures [1]. It represents a major public health concern [2], particularly among individuals aged 50 years and older, with more than half of this population affected by either osteoporosis or low bone mass [1]. Currently, diagnosis primarily relies on dual-energy X-ray absorptiometry (DEXA) for BMD assessment, while treatment typically involves pharmacological agents such as bisphosphonates and selective estrogen receptor modulators (SERMs) [3]. However, these approaches have notable limitations, including the inability of DEXA to detect early-stage bone changes and the potential for adverse effects associated with long-term pharmacotherapy. Moreover, existing treatments often do not fully target the underlying pathophysiological mechanisms of the disease. Therefore, there is an urgent need for the development of novel diagnostic tools and therapeutic strategies that enable earlier detection and offer more effective and targeted interventions.

The COVID-19 pandemic presents an added challenge for individuals with osteoporosis. First, the systemic inflammatory response induced by SARS-CoV-2 infection [4] can adversely affect bone metabolism. Proinflammatory cytokines, such as IL-6 and TNF-α [5], increase the activity of osteoclasts, leading to heightened bone loss. Secondly, certain medications used in the treatment of COVID-19, such as corticosteroids [6], may exacerbate the risk of osteoporosis when administered over prolonged periods. Finally, osteoporosis predominantly affects older adults and individuals with chronic comorbidities—populations that are already at higher risk for severe COVID-19 due to compromised immune function [7]. Moreover, patients with osteoporosis are more susceptible to fractures, which may be further precipitated by complications of COVID-19 such as persistent coughing or falls resulting from postinfection weakness. Therefore, there is a pressing need to develop effective strategies for the management and treatment of osteoporosis in patients affected by COVID-19.

Bioinformatics is an interdisciplinary field that integrates biology, computer science, and information technology to analyze and interpret complex biological data, aiming to uncover the underlying mechanisms of biological processes (BPs) [8]. This discipline provides powerful tools for analyzing large-scale genomic and proteomic datasets, thereby facilitating the identification of disease-specific biomarkers and therapeutic targets [9, 10]. In recent years, bioinformatics has been increasingly applied in the diagnosis, classification, and prognosis prediction of various diseases, including cancer [11, 12].

In the present study, we first identified differentially expressed genes (DEGs) from two gene expression profiles, GSE164805 (osteoporosis) and GSE230665 (COVID-19), and then determined the common DEGs shared between the two conditions. Subsequent analyses included functional enrichment and pathway analysis to explore the biological roles of these DEGs. Furthermore, a protein–protein interaction (PPI) network was constructed to identify key regulatory hub genes. Finally, potential transcription factors (TFs) regulating these hub genes were explored, and candidate therapeutic drugs targeting the hub genes were identified.

## 2. Materials and Methods

### 2.1. Data Collection

To explore the shared molecular mechanisms between osteoporosis and COVID-19, two publicly available gene expression datasets were selected from the Gene Expression Omnibus (GEO) database. The GSE164805 dataset comprises 12 blood samples from osteoporosis patients and 3 from healthy controls, analyzed using the GPL10332 platform (Agilent-026652 Whole Human Genome Microarray 4x44K v2). This dataset provides insights into gene expression changes associated with osteoporosis. Conversely, the GSE230665 dataset includes 10 blood samples from COVID-19 patients and 5 from healthy controls, analyzed on the GPL26963 platform (Agilent-085982 Arraystar human lncRNA V5 microarray). This dataset captures gene expression alterations due to SARS-CoV-2 infection. Comparative analysis of these datasets was conducted to identify common DEGs that may indicate overlapping pathogenic mechanisms.

### 2.2. Data Processing

DEGs were identified using the online tool GEO2R [13]. Genes were considered significantly differentially expressed based on the following criteria: |log2 fold change| > 1 and an adjusted *p* value < 0.05. These thresholds ensured both statistical significance and biological relevance. To identify DEGs commonly dysregulated in both osteoporosis and COVID-19, Venn diagrams were generated using online visualization tools, allowing for the extraction of overlapping gene sets.

### 2.3. Functional Enrichment Analysis

To understand the biological significance of the identified DEGs, we performed functional enrichment analysis using the DAVID 6.8 database [14].

Gene Ontology (GO) analysis was conducted to categorize the DEGs into BPs, cellular components (CCs), and molecular functions (MFs), revealing the roles these genes may play in the cell. Additionally, Kyoto Encyclopedia of Genes and Genomes (KEGG) pathway analysis was performed to identify enriched pathways, providing insight into the biological pathways that are potentially disrupted in both conditions. A significance threshold was set at *p* < 0.05 to focus on the most relevant biological functions and pathways.

### 2.4. PPI Network Analysis

To explore the interactions between the DEGs and understand how they might contribute to disease mechanisms, we constructed a PPI network using the STRING database (https://string-db.org) [15]. This network provides insights into functional associations and interaction patterns among proteins. The resulting PPI network was visualized using Cytoscape software, and the cytoHubba plugin was utilized to identify the top 10 hub genes based on their node degree. Hub genes represent highly interconnected nodes within the network and are often central to disease-related BPs, making them promising candidates for further functional and therapeutic studies.

### 2.5. TF–Hub Gene Networks

Understanding the regulation of hub genes is essential for comprehending disease mechanisms. We used the NetworkAnalyst platform [16] to identify TFs that regulate these hub genes. A TF–hub gene regulatory network was subsequently constructed to illustrate potential regulatory interactions. This analysis helps identify key transcriptional regulators that may contribute to the shared pathophysiology of osteoporosis and COVID-19 and could serve as novel biomarkers or therapeutic targets.

### 2.6. Drug–Gene Interaction

To identify potential therapeutic agents, we investigated existing drugs or compounds that interact with the hub genes using the Drug–Gene Interaction Database (DGIDB) [17]. This analysis aimed to repurpose known drugs for the treatment of osteoporosis patients infected with COVID-19 by targeting shared molecular pathways. Identifying such drugs can accelerate the development of effective treatments by leveraging existing pharmacological knowledge.

## 3. Results

### 3.1. Identification of DEGs

**Figure 1:**
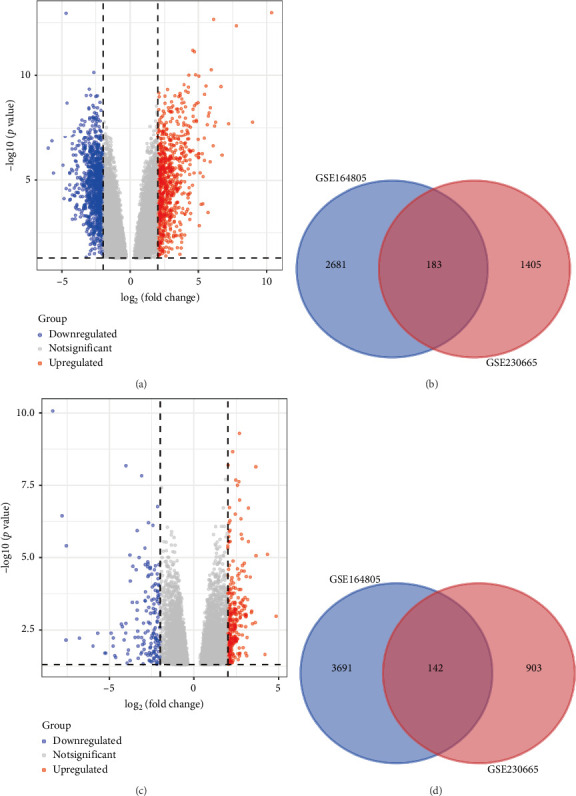
Identification of common differentially expressed genes (DEGs) for COVID-19 patients and osteoporosis. (a) Volcano plots of the DEGs in GSE164805. (b) 183 common DEGs with upregulation between COVID-19 patients with osteoporosis. (c) Volcano plots of the DEGs in GSE230665. (d) 142 common DEGs with downregulation between COVID-19 patients with osteoporosis.

Using the defined criteria, we identified a total of 325 shared DEGs between the osteoporosis and COVID-19 datasets, comprising 183 upregulated and 142 downregulated genes (Figure 1). The identification of these common DEGs suggests that there are overlapping molecular mechanisms and pathways affected in both conditions. This finding provides a foundation for further analysis to understand how COVID-19 may exacerbate osteoporosis and vice versa.

### 3.2. GO and KEGG Pathway Enrichment Analysis

Functional enrichment analysis of the shared DEGs revealed significant BPs and pathways. GO analysis showed enrichment in categories such as “signal transduction” and “phosphorylation,” indicating alterations in cell signaling mechanisms. The enrichment in “plasma membrane” components suggests changes at the cellular interface, which may affect cell communication and transport. “Metal ion binding” was also significant, highlighting potential disruptions in metal ion homeostasis, which is important for bone health.

**Figure 2:**
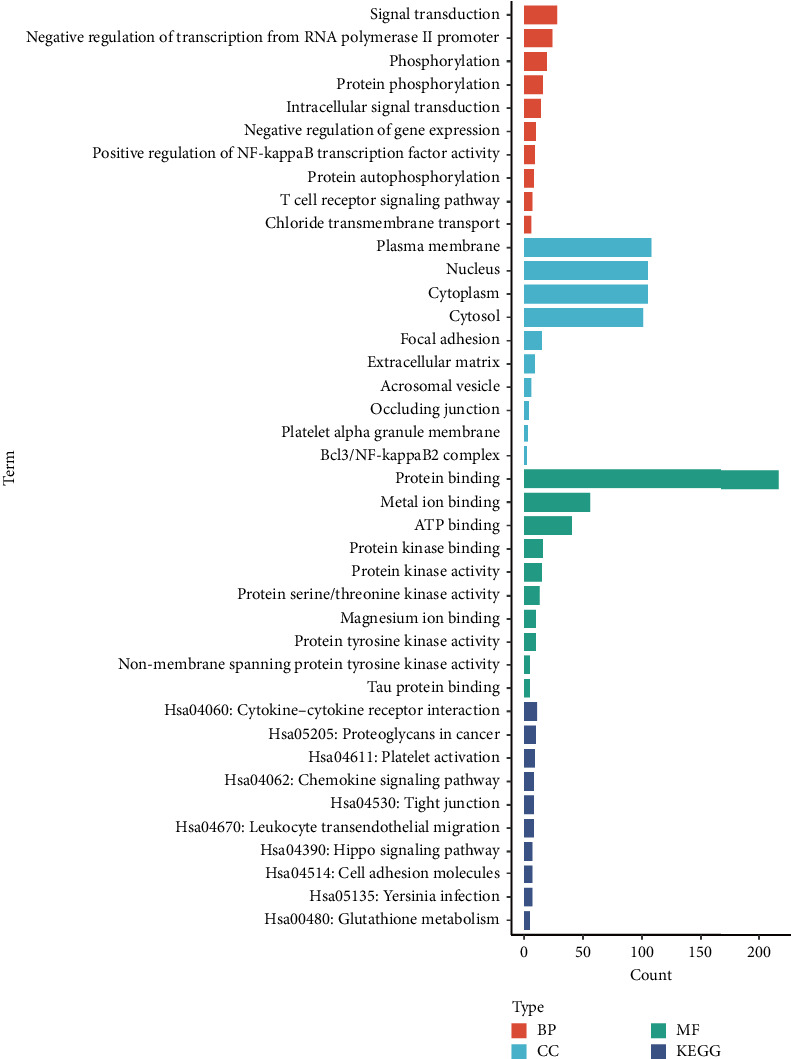
Functional analyses including biological process (BP), molecular function (MF), and cellular component (CC) and KEGG pathway analyses, separately.

KEGG pathway analysis identified significant enrichment in pathways like “Cytokine–cytokine receptor interaction,” suggesting an involvement of immune signaling in both diseases. The “Proteoglycans in cancer” pathway enrichment may indicate changes in extracellular matrix components, affecting bone structure. The “Hippo signaling pathway” is crucial for regulating cell proliferation and apoptosis, and its alteration could contribute to disease progression (Figure 2). These results provide insights into the MFs and pathways that may be commonly dysregulated in osteoporosis and COVID-19.

### 3.3. PPI Network

**Figure 3:**
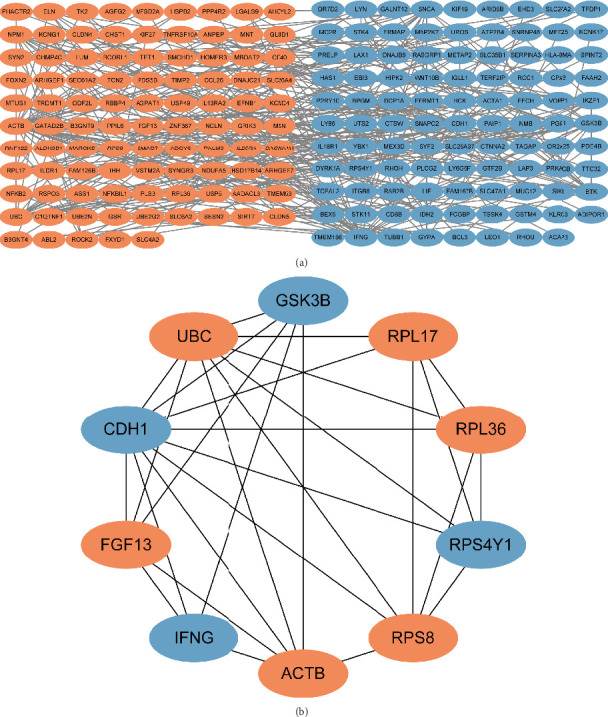
Protein–protein interaction network (a) and hub genes (b). Orange stands for upregulation gene, while blue for downregulation.

The PPI network constructed from the shared DEGs consisted of 193 nodes (genes) and 397 edges (interactions) (Figure 3(a)). The network visualization helps in understanding the complex interplay between different proteins. Using the cytoHubba plugin, we identified the top 10 hub genes: ACTB, CDH1, RPS8, interferon gamma (IFNG), RPL17, UBC, RPL36, RPS4Y1, GSK3B, and FGF13 (Figure 3(b)). These hub genes have high connectivity and may play pivotal roles in the shared pathogenesis of osteoporosis and COVID-19.

### 3.4. TF–Hub Gene Networks

**Figure 4:**
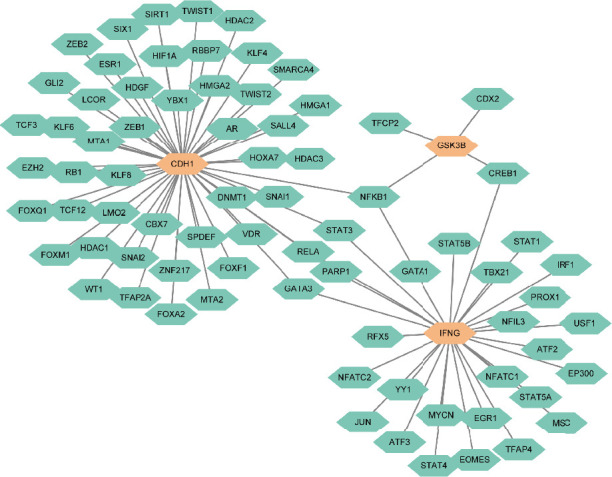
The hub gene and transcription factor (TF) interaction network. Orange represents hub genes; cyan indicates TFs.

To further understand the regulation of these hub genes, we identified 76 TFs that potentially regulate them (Figure 4). The TF–hub gene network provides insights into the upstream regulatory mechanisms that may lead to altered gene expression in both diseases.

### 3.5. Drug–Gene Interaction

Our analysis identified 15 drugs or compounds targeting four of the hub genes: ACTB, CDH1, GSK3B, and IFNG (Table 1). The identification of these drug–gene interactions suggests potential candidates for drug repurposing, offering a promising avenue for developing treatments for osteoporosis patients affected by COVID-19.

## 4. Discussion

The ongoing COVID-19 pandemic has introduced additional challenges for individuals with preexisting chronic conditions, including osteoporosis. In this study, we employed a comprehensive bioinformatics approach to explore the molecular overlap between osteoporosis and SARS-CoV-2 infection, aiming to identify shared DEGs, key signaling pathways, hub regulatory genes, and potential therapeutic targets that may inform future diagnostic or treatment strategies.

Our results identified 325 shared DEGs, including 183 upregulated and 142 downregulated genes, highlighting common molecular pathways potentially influenced by both osteoporosis and COVID-19. The KEGG pathway analysis revealed significant enrichment in pathways such as Hippo signaling pathway. The Hippo signaling pathway is extensively studied for its roles in osteoporosis and COVID-19. The Hippo signaling pathway is an evolutionarily conserved cascade that regulates organ size and tissue homeostasis by controlling cell proliferation, apoptosis, and stem cell self-renewal [18]. Its core components include the kinases MST1/2 and LATS1/2, along with the downstream transcription coactivators YAP and TAZ [19]. These coactivators interact with TEAD family TFs to regulate gene expression [20]. Dysregulation of this pathway has been implicated in various diseases, including cancer and metabolic disorders. Notably, recent studies have also linked Hippo signaling to both osteoporosis and viral infections. During SARS-CoV-2 infection, activation of the Hippo pathway contributes to the host's antiviral response, and inhibition of upstream kinases MST1/2 and LATS1 enhances viral replication, indicating its protective role against infection [21].

Further network analysis identified 10 hub genes—ACTB, CDH1, RPS8, IFNG, RPL17, UBC, RPL36, RPS4Y1, GSK3B, and FGF13—that appear to be central to the PPI network. These genes are likely involved in key BPs shared between osteoporosis and COVID-19. Importantly, drug–gene interaction analysis using the DGIDB revealed 15 FDA-approved drugs targeting four of these hub genes (ACTB, CDH1, GSK3B, and IFNG), highlighting the potential for drug repurposing in this patient population. This strategy may accelerate the development of effective therapies by leveraging existing pharmacological knowledge and reducing the time required for clinical translation.

ACTB is a crucial protein in maintaining cellular structure and function [22]. Its role in bone cell activity links it to osteoporosis [23], while its involvement in cytoskeletal dynamics and immune responses connects it to the pathophysiology of COVID-19. Osteoclasts rely on actin cytoskeleton for their bone-resorbing activity. Abnormalities in ACTB expression or function can impair osteoclast activity, leading to imbalances in bone resorption and formation, which is a hallmark of osteoporosis [24]. ACTB is involved in the regulation of immune cell movement and function. During COVID-19, a dysregulated immune response can lead to severe inflammation. Understanding the role of ACTB in these processes could provide insights into managing COVID-19–related inflammation [25].

CDH1, also known as E-cadherin, is a protein that plays a crucial role in cell–cell adhesion in epithelial tissues [26]. It is essential for maintaining the integrity and structure of epithelial layers [27]. Dysregulation of CDH1 is often associated with cancer progression and metastasis. CDH1 has been shown to regulate osteoblast function through an APC/C-independent modulation of Smurf1. This suggests that modulation of Cdh1 could be a potential therapeutic option for treating osteoporosis [28]. Inflammatory responses and immune cell activation are key features of severe COVID-19. CDH1, as a cell adhesion molecule, may indirectly influence the immune response and the severity of inflammation through its role in cellular interactions and tissue integrity [29].

GSK3B is a serine/threonine kinase involved in various cellular processes, including metabolism, cell proliferation, and apoptosis [30]. GSK3B's activity is tightly regulated by phosphorylation, and its dysregulation is associated with multiple diseases, including diabetes [31] and cancer [32]. GSK3B plays a significant role in bone metabolism by regulating osteoblast and osteoclast activities. It affects osteoblast differentiation and bone formation through the Wnt/β-catenin signaling pathway. Inhibition of GSK3B enhances osteoblast function and bone formation [33]. GSK3B is essential for the phosphorylation of the nucleocapsid protein of SARS-CoV-2, which is crucial for viral replication. Inhibitors of GSK3B, such as lithium, have been shown to reduce the replication of SARS-CoV-2 by blocking this phosphorylation process [34]. Research indicates that targeting GSK3B might offer a dual benefit in COVID-19 treatment by inhibiting viral replication and boosting the immune response. This approach could help in controlling the infection and improving patient outcomes [35].

IFNG is a cytokine critical for innate and adaptive immunity against viral and intracellular bacterial infections and for tumor control [36]. It is produced primarily by natural killer (NK) and natural killer T (NKT) cells as part of the innate immune response and by CD4+ and CD8+ T cells once the adaptive immune response is engaged. IFNG can increase bone resorption by enhancing osteoclast activity. Studies indicate that elevated levels of IFNG are associated with increased osteoclastogenesis, which leads to enhanced bone resorption and contributes to the development of osteoporosis, particularly in postmenopausal women [37]. Inflammatory cytokines such as IL-1 beta, IL-6, and TNF-alpha have been shown to be elevated in osteoporotic patients, while IFNG levels do not show significant differences [38]. This suggests a complex interplay between different cytokines in bone remodeling and osteoporosis progression. IFNG plays a critical role in the immune response to SARS-CoV-2, the virus causing COVID-19. Severe cases of COVID-19 are often characterized by a cytokine storm, where excessive levels of proinflammatory cytokines, including IFNG, contribute to severe lung damage and multiorgan failure. Elevated IFNG levels are part of this hyperinflammatory response, which can exacerbate the severity of the disease [39]. Genetic polymorphisms in IFNG have been studied for their potential impact on the severity of COVID-19. Certain variants may influence the expression and activity of IFNG, thereby affecting individual susceptibility to severe outcomes in COVID-19 [40].

The regulatory network analysis identified 76 TFs that interact with these hub genes. This significant interplay between TFs indicates complex regulatory mechanisms that control gene expression in osteoporosis and COVID-19. TFs are proteins that bind to specific DNA sequences to control the transcription of genetic information from DNA to messenger RNA. They play a crucial role in regulating gene expression and are involved in various cellular processes, including development, cell proliferation, and response to environmental stimuli. Estrogen receptors (ERs), which are TFs, significantly influence bone homeostasis. Polymorphisms in the ER genes can affect bone density and the risk of osteoporosis [41]. TFs such as NF-κB and STAT3 are crucial in regulating the immune response to SARS-CoV-2 infection. NF-κB modulates the expression of proinflammatory cytokines, which are elevated in COVID-19, contributing to the cytokine storm observed in severe cases [42]. Specific TFs have been identified to alter gene expression patterns in COVID-19 patients, impacting disease severity. For example, TFs like STAT and E2F/MYB are associated with poor outcomes in severe COVID-19 due to their role in regulating inflammatory responses [43].

The DGIDB database serves as a publicly accessible repository that focuses on amalgamating and presenting the interactions between drugs and genes. By aggregating data from various sources, the database offers insights into which drugs interact with specific genes. This information is crucial for drug development, gene function investigation, and personalized medicine [44]. In our prior studies, we utilized the DGIDB database to identify drugs promoting blood coagulation that could potentially serve as targets for treating pancreatic cancer [45]. In the current study, these repurposed drugs may offer new treatment options for osteoporotic patients who are at higher risk of complications from SARS-CoV-2 infection.

Despite the strengths of our bioinformatics approach, several limitations should be acknowledged. First, this study is based solely on publicly available transcriptomic datasets and lacks experimental validation of the identified DEGs, hub genes, and drug interactions. Functional studies are needed to confirm the biological relevance of the predicted molecular mechanisms and therapeutic targets. Second, the sample sizes of the included datasets are relatively small, which may limit the statistical power and generalizability of our findings. Third, the heterogeneity in platforms and methodologies used across the datasets could introduce biases in differential expression analysis. Finally, while drug repositioning offers a promising avenue for rapid therapeutic development, clinical trials are essential to assess the safety and efficacy of these agents in the specific context of osteoporosis and SARS-CoV-2 comorbidity.

## 5. Conclusion

This study successfully identified 15 potential drugs targeting four genes, which may benefit osteoporosis patients with COVID-19. These findings pave the way for targeted therapeutic strategies to mitigate the impact of COVID-19 on osteoporosis patients, ultimately improving their clinical outcomes.

## Figures and Tables

**Table 1:** The candidate drugs and corresponding genes for COVID-19 and osteoporosis.

Gene	Drug
ACTB	Ethinyl estradiol
CDH1	Bicalutamide
GSK3B	Methotrexate
IFNG	Amikacin
IFNG	Bleomycin
IFNG	Ganciclovir
IFNG	Ibuprofen, sodium salt
IFNG	Interferon alfa-2B
IFNG	Methylprednisolone
IFNG	Pefloxacin
IFNG	Prednisone
IFNG	Rituximab
IFNG	Theophylline
IFNG	Thrombin
IFNG	Ursodiol

## Data Availability

The datasets used and/or analyzed during the current study are available from the corresponding authors on reasonable request.
